# KK-LC-1 may be an effective prognostic biomarker for gastric cancer

**DOI:** 10.1186/s12885-021-07974-7

**Published:** 2021-03-12

**Authors:** Jun Ji, Jiahui Chen, Anqiang Wang, Wei Zhang, Hongge Ju, Yang Liu, Leping Li

**Affiliations:** 1 Department of Gastrointestinal Surgery, Shandong Provincial Hospital, Cheeloo College of Medicine, Shandong University, Jinan, 250021 Shandong China; 2 First Affiliated Hospital of Baotou Medical College, General Surgery, Baotou, 014010 Inner Mongolia China; 3 Department of Gastrointestinal Surgery, Key Laboratory of Carcinogenesis and Translational Research (Ministry of Education), Peking University Cancer Hospital & Institute, Beijing, 100142 China; 4 Baotou Medical College, Inner Mongolia University of Science & Technology, Baotou, 014060 Inner Mongolia China; 5 Department of Gastrointestinal Surgery, Shandong Provincial Hospital Affiliated to Shandong First Medical University, Jinan, 250021 Shandong China

**Keywords:** Gastric cancer, KK-LC-1, Overall survival, Risk score, Biomarker

## Abstract

**Background:**

The objective of the study was to detect the expression of Kita-Kyushu lung cancer antigen-1 (KK-LC-1) in gastric cancer (GC) specimens and analyse the associations between KK-LC-1 expression and clinicopathological parameters and clinical prognosis.

**Methods:**

All of the 94 patients in this study were GC patients who underwent surgical resection. KK-LC-1 protein expression in GC tissue was detected by immunohistochemistry. This report applies the histological score (H-score) to evaluate KK-LC-1 expression. To calculate this indicator, the number of positive cells in each section and their staining intensity were converted to corresponding values. The expression of KK-LC-1 in the cytoplasm of cancer and normal tissues was scored to obtain their respective H values. The chi-square test, Kaplan-Meier method and Cox regression were used to analyse the linear association between KK-LC-1 expression and clinicopathological data and prognosis.

**Results:**

In the cytoplasm, KK-LC-1 expression in tumour tissues was significantly higher than that in normal tissues (*P* < 0.001). Using the median H-score as the cut-off value, we discovered that GC patients with high levels of KK-LC-1 expression in the cytoplasm had favourable overall survival (OS) (*P* = 0.016), and this result was statistically significant in the Cox regression analysis. Additionally, a negative correlation was found between KK-LC-1 protein expression and the pathological grade of the tumour (*P* = 0.036), with significantly more KK-LC-1 protein expression observed in the intestinal type of GC than in the diffuse type (*P* = 0.008).

**Conclusions:**

Our research data showed that KK-LC-1 expression was greater in GC tissues than in normal tissues, and higher KK-LC-1 expression was associated with longer OS of GC patients. KK-LC-1 can be used as a biomarker for a good prognosis in GC patients.

## Background

Gastric cancer (GC) is the third leading cause of cancer-related death worldwide. Most patients with gastric cancer are usually not diagnosed until the late stages of the disease, so the prognosis is usually poor. The 5-year survival rate of all patients, including surgical patients, does not exceed 30% [1]. It has been suggested that tumour tissue type, TNM stage and patient physical and mental conditions are all important factors affecting the treatment of GC [2]. Metastatic GC has few treatment options, and the treatment goal is palliative rather than therapeutic. Although a number of chemotherapy drugs have been shown to be effective in treating gastric cancer, one fact we cannot ignore is that there are limitations to using targeted therapy for GC, which mainly targets the vascular endothelial growth factor (VEGF) pathway and HER2. Recent research and information regarding the genetic background of GC may give us more opportunities for targeted therapy [3].

In addition, various molecular biomarkers are efficient diagnostic and prognostic tools for gastric cancer, but these biomarkers need further validation before they can be used in daily clinical practice. At present, the only biomarkers used for GC are carcinoembryonic antigens CA 19–9, CA-50 [4] and CA-72 [5]. However, these are limited by insufficient sensitivity and specificity to evaluate GC diagnosis and prognosis, and the efficacy of targeting these biomarkers for clinical treatment is doubtful.

There are already some prognostic markers for GC. For example, studies have shown that the *Ki-67* index has significance for the prognosis of cancer [6]. Therefore, the high expression of *Ki-67* can be used to predict poor prognosis in GC patients. Meta-analysis and systematic reviews of the literature indicate that MSI-H and EBV-positive gastric cancers are usually associated with improved prognosis and prolonged survival [7]. Low expression of *miR-433* and high expression of *miR214* are independent predictors of poor prognosis [8]. In addition, a large number of studies have shown that *c-MET* overexpression is associated with poor survival prognosis [9–11]. However, the number of effective prognostic markers for GC is still very small, and there is a need to identify novel and effective biomarkers to determine the prognosis of GC and to establish new treatment approaches.

Cancer-testis antigens (CTA) are characterized by their spontaneous immunogenicity and unique expression pattern. CTAs are normally expressed only in the germ cells of the normal human testis and placenta, but are also activated in tumour cells [12, 13]. T cells and anti-CTA protein antibodies can be detected in cancer patients [14–18], suggesting that abnormal expression of CTA can induce tumour tissues to produce an adaptive immune response. Since CTAs are tumour-specific, they are believed to be potential effective targets for new therapeutic strategies, such as immunotherapy [19, 20]. At the same time, CTA expression in several types of cancer has potential significance for prognosis [21]. Although CTA expression has been studied in many cancers, few studies have focused on gastric cancer [21–23]. Kita-Kyushu lung cancer antigen-1 (KK-LC-1) is also known as *CT83*. Futawatari N et al. found that in early stages of GC, high CT83 expression rates can be frequently detected [24].

Previous research by our team has shown that KK-LC-1 mRNA expression is related to the prognosis of gastric cancer [25]. Therefore, we studied KK-LC-1 protein expression in GC specimens and also analysed the relationship between KK-LC-1 protein expression and clinicopathological parameters and prognosis.

## Methods

### Tissue microarrays

Tissue arrays containing multiple human gastric cancer tissues (HStm-Ade180Sur-17) were obtained from Shanghai Outdo Biotech. The tissue chip samples were all gastric adenocarcinomas. The samples included 94 gastric cancer tissues and 84 adjacent tissues. The operation time was May 2007 to February 2008, and the date of last follow-up was July 2015. The diameter of each sample spot was 1.5 mm, and the thickness of the tissue section was 4 μm. The EnVision+ detection system (Dako) was used per the manufacturer’s instructions. From these tissue arrays, 85 pairs of GC specimens and corresponding adjacent normal tissue specimens were obtained, as well as ten individual cancer tissue specimens. Surgical type was categorized as curative or noncurative resection. Radical resection (R0) refers to the complete removal of the tumour, no residue under the microscope, and complete removal to the naked eye but residual tumour under the microscope (R1) or visible both by the naked eye and pathology (R2), are considered noncurative from the patient’s medical record, and the patient was followed up with since the surgery date. The GC patients were staged according to the 7th edition of the American Joint Committee on Cancer staging manual. The end point of follow-up is overall survival (OS), which refers to the interval between the date of surgery and cancer-related death. This study was approved by the Ethics Committee of Shanghai Outdo Biotech Company.

### Immunohistochemistry and H-scoring of KK-LC-1

Immunohistochemical staining was performed manually, and each slide was carefully handled in strict accordance with the instructions. Anti-KK-LC-1 antibody (CL4762, Abcam, United Kingdom, 100 μL), produced in mice, was applied in the expression analysis. Immunohistochemical slide staining results were independently evaluated by two experienced pathologists. The H-scoring system was used to evaluate KK-LC-1 staining results [26–29], and we estimated the H-score by multiplying the total staining intensity of each section by the percentage of the number of positive cells. The staining intensity was divided into 4 levels varying from 0 to 3: 0 represents negative; 1 represents weak; 2 represents medium and 3 represents strong, and the percentage of positives was between 0 and 100. In general, the final H-score obtained was between 0 and 300. We stained KK-LC-1 in the cytoplasm and nucleus separately and obtained their respective values by scoring.

### Statistical analysis

This study used SPSS 17.0 software for statistical analysis. To evaluate sample distribution, the Kolmogorov-Smirnov nonparametric test was applied. The Mann-Whitney U test was used to compare variables with abnormal distribution, and the chi-square test was used to compare qualitative variables. OS was compared with the Kaplan-Meier method and log rank test. All potential factors related to prognosis from univariate analysis were input into the Cox regression model. To identify independent prognostic factors, this paper applies multifactor Cox regression analysis. All *P*-values were bidirectional, and *P*-values that were less than 0.05 were considered to be statistically significant.

## Results

### Clinicopathological characteristics and survival data

The average and median ages of patients undergoing gastric cancer surgery were 65 and 66 (range 45–83 years old), respectively. Male patients comprised 75.5% of the study group, and the ratio of male to female patients was 3:1. Ninety-five percent of the patients underwent radical resection, of which moderately to highly differentiated adenocarcinoma was found in 24.4% of cases. Lymph node metastasis and distant metastasis was found in 21.2 and 4% of patients, respectively. Table 1 lists more information about this result.

**Table 1:** Clinicopathological characteristics of the tissue microarrays

Characteristic	n (%)	Characteristic	n (%)
Age (yr)		N2	22 (23.4)
≤ 65	44 (46.5)	N3a	29 (30.9)
>65	50 (53.2)	N3b	6 (6.4)
Sex		Pathological grades	
Female	23 (24.5)	I	23 (24.5)
Male	71 (75.5)	II-III	13 (13.8)
Tumor Size (cm)		III	50 (53.2)
≤ 5	38 (40.4)	III-IV	8 (8.5)
>5	56 (59.6)	Pathological Stage	
T Stage		IA	2 (2.1)
T1a	1 (1.1)	IB	4 (4.3)
T1b	2 (2.2)	IIA	13 (13.8)
T2	9 (9.6)	IIB	15 (16.0)
T3	62 (66.0)	IIIA	22 (23.4)
T4a	18 (19.1)	IIIB	29 (30.9)
T4b	2 (2.1)	IIIC	5 (5.3)
M Stage		IV	4 (4.3)
M0	90 (95.7)	Survival data (year)	
M1	4 (4.3)	≤3	58 (62%)
N0	20 (21.3)	>3	36 (38%)
N1	17 (18.1)		

### Staining and H-scoring for KK-LC-1 expression

**Fig. 1:**
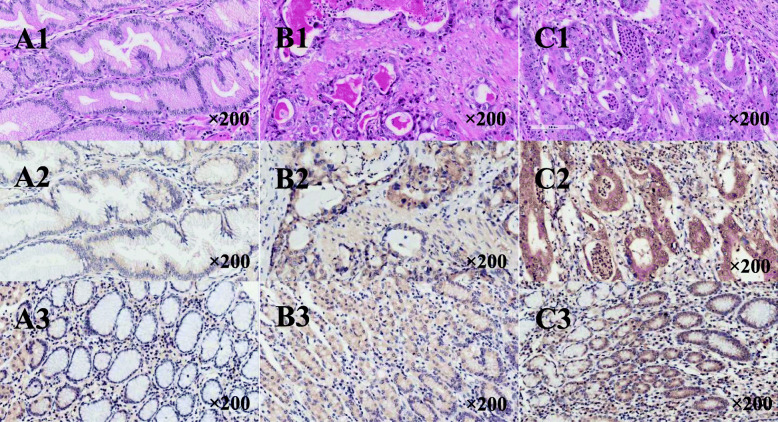
HE and Immunohistochemical staining of tissues (magnification, × 200). A1、B1 and C1: HE staining; A2、B2 and C2: Immunohistochemical staining of cancer tissue, with< 1, 1 and 2 intensity, respectively; A3、B3 and C3: Immunohistochemical staining of adjacent tissue, with< 1, 1 and 1.5 intensity, respectively. A1、A2 and A3 are the same sample, B1、B2 and B3 are the same sample, C1、C2 and C3 are the same sample

**Fig. 2:**
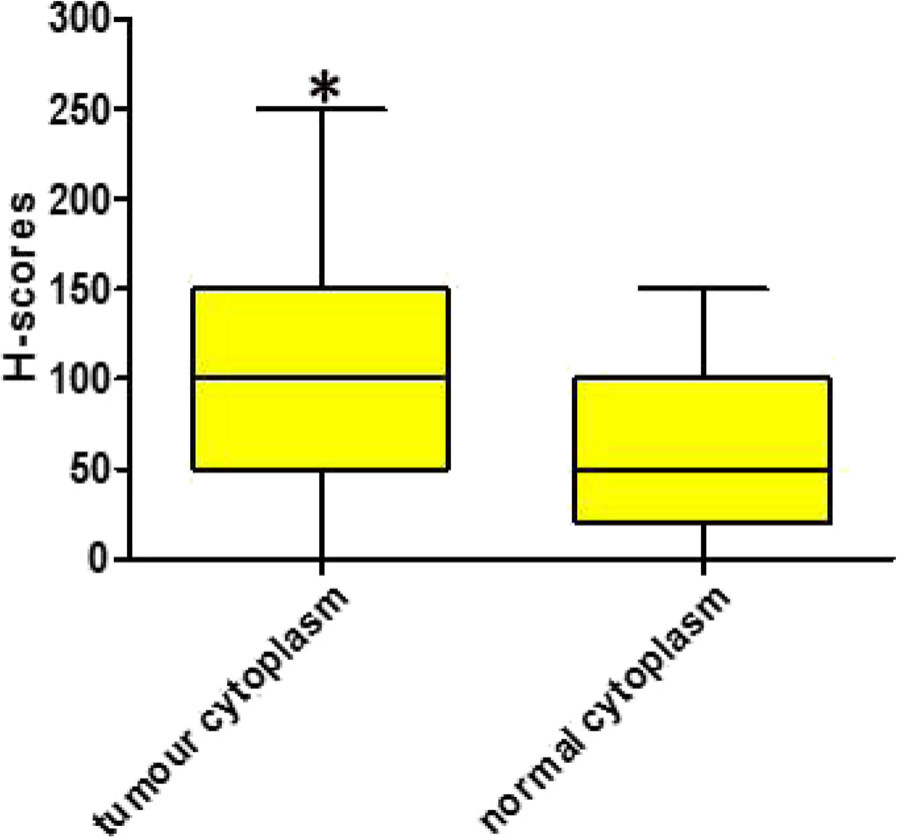
Box plot of Kita-Kyushu lung cancer antigen-1 H scores in tumors and normal tissues. *Indicates that there is a significant difference between the two groups, and the *P* < 0.001

KK-LC-1 staining was quantified and analysed (Table 2 and Fig. 1). In the tissue arrays, all stained specimens were located in the cytoplasm, and no strong positive (3+) staining was found in the specimens. The H-scores of tumour tissue and normal tissue were calculated separately, and the empirical results are reported in Table 3 and Fig. 2. We found that the median H-score of KK-LC-1 in the cytoplasm of tumour tissue was 100 (range 0–250). In normal tissues, we calculated that the median H score of KK-LC-1 was 50 (range 0–150).

**Table 2:** Kita-Kyushu lung cancer antigen-1 staining results in tumor and normal tissues

Cytoplasmic staining	Positive cell rate (%) Median (range)		***n*** (%)	
	Tumor tissues	Normal tissues	Tumor tissues	Normal tissues
Negative	–	–	4 (4.3)	8 (9.5)
Positive	100 (40–100)	90 (10–100)	90 (95.7)	76 (90.5)
< 1	90 (80–100)	60 (10–100)	19 (20.2)	36 (42.9)
1+	100 (40–100)	90 (20–100)	57 (60.7)	40 (47.6)
2+	100 (90–100)	–	14 (14.8)	0 (0)

Negative = 0, Positive: 1≦1+ < 2, 2≦2+ < 3.

**Table 3:** H-score values in tumor and normal tissues

Group	***n***	Localization	Minimum	Maximum	Median
Tumor	94	Cytoplasm	0	250	100
Normal	84	Cytoplasm	0	150	50

### Comparisons and cut-off values of KK-LC-1 expression

In both tumour and normal tissues, KK-LC-1 was found to be expressed only in the cytoplasm. As shown in the results of the Kolmogorov-Smirnov test, the H score values were abnormally distributed. Therefore, we chose the Mann-Whitney U test to analyse the data (Table 4). In normal tissues and tumour tissues, KK-LC-1 is expressed only in the cytoplasm. At the same time, we observed that KK-LC-1 expression was higher in tumour tissues than in normal tissues (*P* < 0.001).

**Table 4:** Comparison in tumor and normal tissues for Kita-Kyushu lung cancer antigen-1 expression

Group	***n***	Minimum	Maximum	Median	Mean rank	***P***
Tumor	94	0	250	100	111.00	< 0.001^a^
Normal	84	0	150	50	65.44	

^a^Statistically significant

**Fig. 3:**
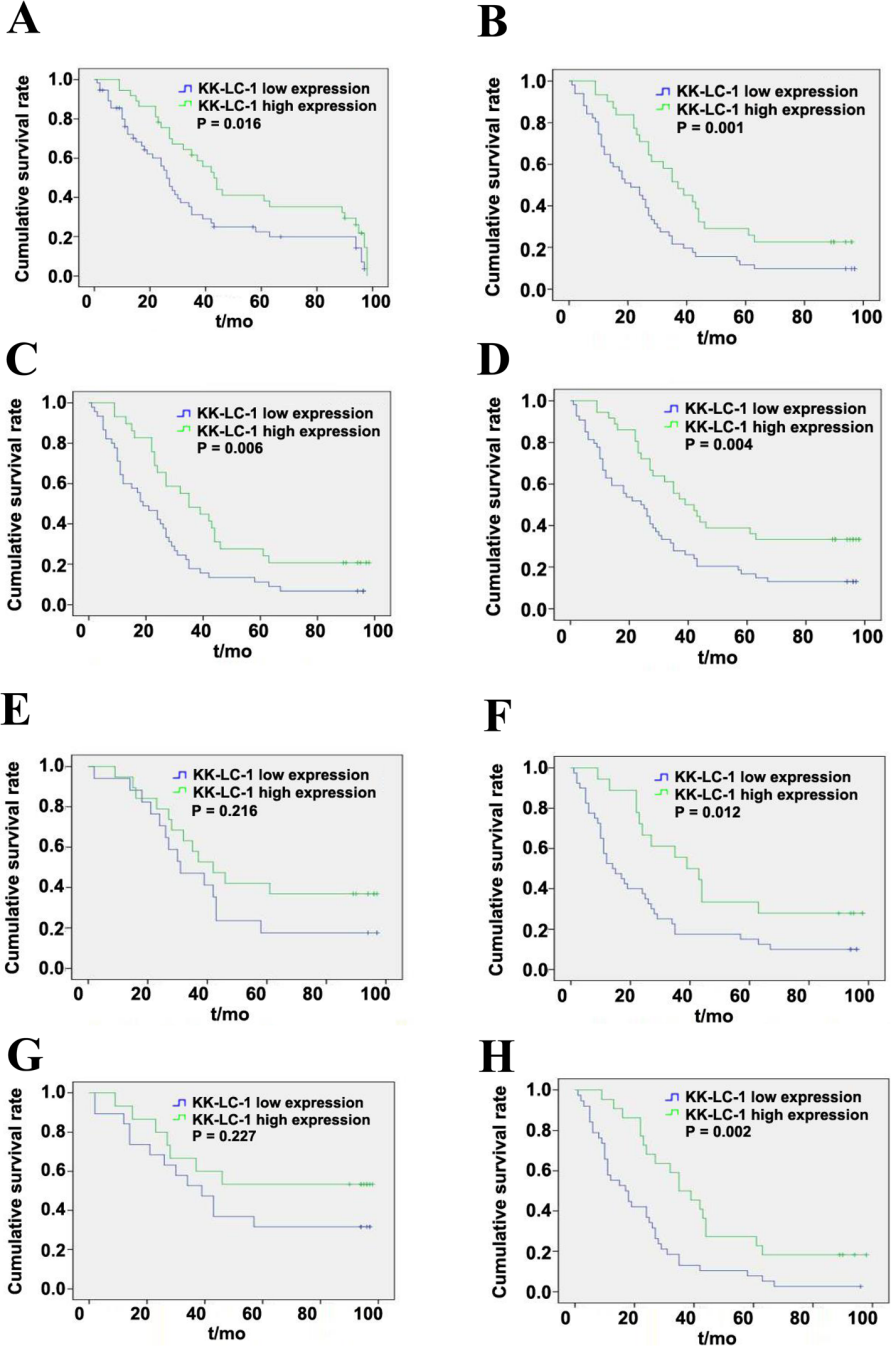
Kaplan-Meier survival analyses in different subgroups, according to Kita-Kyushu lung cancer antigen-1 expression. **a**: The whole cohort; **b**: T3 + 4 group; **c**: Positive Nodal involvement group; **d**: M0 group; **e**: Pathological grades <III group; **f**: Pathological grades ≥III; **g**: Pathological Stage I + II group; **h**: Pathological Stage III + IV group. KK-LC-1: Kita-Kyushu lung cancer antigen-1

Due to the difference in the median H score of tissue staining, all GC patients were divided into two subsamples. Univariate and multivariate analysis results indicated that favourable OS (as displayed in Fig. 3a) was generally associated with high H-scores in the cytoplasm. Univariate survival analysis (Table 5) showed that KK-LC-1 expression (*P* = 0.016), T stage (*P* = 0.002), N stage (*P* = 0.001) and clinical stage (*P* < 0.001) were associated with OS in GC. In our study, age, sex, tumour size, M stage and pathological grade had no significant effect. The empirical results of CoS regression analysis showed that T stage, N stage and higher expression of KK-LC-1 protein were independent prognostic factors of OS (Table 5).

**Table 5:** Univariate and multivariate analyses for Gastric cancer

Univariate	χ^**2**^	***P*** value	***OR*** (95%CI)
Age	0.577	0.565	
Sex	0.871	0.351	
Tumor Size	2.632	0.105	
T Stage	9.868	0.002^a^	
N Stage	11.973	0.001^a^	
M Stage	1.873	0.053	
Pathological grades	3.753	0.171	
Pathological Stage	10.464	0.001^a^	
Protein Expression	5.848	0.016^a^	
Multivariate			
T Stage		0.007^a^	3.466 (1.394–8.622)
N Stage		0.009^a^	2.986 (1.308–6.814)
Protein Expression		0.034^a^	0.591 (0.364–0.961)

^a^ Statistically significant

### Correlations between KK-LC-1 expression and clinicopathological parameters

In Table 6, we marked the association between KK-LC-1 expression and the clinicopathological data in detail. KK-LC-1 expression was not significantly associated with age, sex, tumour size, TNM stage or clinical stage. However, patients with a low pathological grade and those with intestinal-type GC exhibited relatively high expression of the KK-LC-1 protein, and the *P* value was statistically significant.

**Table 6:** Correlation between KK-LC-1 expression and clinicopathological parameters in tumor

Parameter	Low expression	High expression	χ^**2**^	***P*** value
Age				
≤ 65	29	15	0.963	0.326
>65	28	22		
Sex				
Female	15	8	0.268	0.605
Male	42	29		
Tumor Size (cm)				
≤ 5	21	17	0.772	0.380
> 5	36	20		
T Stage				
T1 + 2	6	6	0.652	0.419
T3 + 4	51	31		
M Stage				
M0	54	36	0.361	0.548
M1	3	1		
N Stage				
Negative	12	8	0.004	0.947
Positive	45	29		
Pathological grades				
<III	17	19	4.400	0.036^a^
≥ III	40	18		
Clinical Stage				
I + II	19	15	0.505	0.477
III + IV	38	22		
Histological				
Intestinal type	17	22	7.072	0.008^a^
Diffuse type	39	16		

^a^Statistically significant

## Discussion

In this study, GC patients with higher levels of KK-LC-1 expression were found to have a better prognosis, and the overall expression of KK-LC-1 protein in gastric cancer tissue was higher than that in normal tissues. Fukuyama et al. [30] found similar results: KK-LC-1 gene expression was found to be higher in tumour regions than in non-tumour regions, and KK-LC-1 was found to be expressed in non-tumour sites carrying stomach tumour tissue. In our experimental findings, the KK-LC-1 protein expression rate in tumour tissues was 95.7%. In contrast, Akiko et al. found that the gene expression rate of KK-LC-1 reached 81.6%, which was significantly higher than that found in other studies [23]. One study found that the KK-LC-1 expression rate in triple-negative breast cancer was 75% [31].. These findings are similar to ours, which suggests that KK-LC-1 is likely to be highly expressed in tumours. However, no existing studies have focused on the expression of tumour-associated antigens in gastric cancer as highly expressed as KK-LC-1, suggesting that KK-LC-1 could be an ideal therapeutic target. For clinical diagnostic applications, high expression of tumour-associated antigens in the early stages of cancer is often considered a useful target. At present, there are few reports on KK-LC-1 gene and protein expression and tumour prognosis. Thus, more research is needed for verification.

Generally, this study’s findings suggest that there is a significant negative correlation between KK-LC-1 protein expression and pathological grade. The higher the pathological grade is, the lower the KK-LC-1 protein expression in the tissue and the poorer the prognosis. In contrast, the lower the pathological grade is, the higher the KK-LC-1 protein expression in the tissue and the better the prognosis of the patient. This result also indirectly shows the reliability of our experimental data. We hypothesized that the KK-LC-1 protein is associated with the early stage of the tumour and thus related to a good prognosis. Therefore, KK-LC-1 can be used as a positive biomarker directly related to prognosis and provides clinicians with more choices. For example, patients with higher KK-LC-1 protein expression levels may achieve better results from adjuvant chemotherapy or radiotherapy than patients with lower expression levels. However, studies have also shown that the expression level of KK-LC-1 in hepatocellular carcinoma (HCC) is increased. High KK-LC-1 expression levels are associated with poor survival outcomes in HCC. This study also found that KK-LC-1 promotes cell growth, invasion, migration and epithelial-mesenchymal transition in vivo and in vitro [30]. In summary, abnormal KK-LC-1 protein expression is clearly related to the occurrence and development of tumours. Therefore, KK-LC-1 may play different roles in different malignant tumours, and more in-depth research is required to verify the true relationship between KK-LC-1 and cancer and the specific mechanisms of involvement.

According to the classification proposed by Lauren, GC can be divided into 2 histological types under microscopic examination, namely, the intestinal type and the diffuse type. Intestinal GCs originate from premalignant lesions, initially chronic gastritis caused by *Helicobacter pylori*, followed by atrophic and metaplastic gastritis. However, diffuse GC is directly induced by active inflammation of the gastric mucosa [32, 33]. Generally, the diffuse type has a poor prognosis and a higher risk of lymph node metastasis (LNM), while the intestinal type has a better prognosis [34].. Our experimental results are consistent with this conclusion. KK-LC-1 is highly expressed in the intestinal type, and the prognosis is good.

To date, some mechanisms linking KK-LC-1 and neoplasia have been revealed in several tumours. According to reports, the activation of CT genes in some types of cancer is related to hypomethylation of CpG islands. *CT45* is one of the 6 member families of the X-linked CT gene, and the expression of *CT45* associated with hypomethylation of promoter DNA is increased in epithelial ovarian cancer. Researchers believe that *CT45* expression may be a prognostic biomarker [35].. In lung adenocarcinoma, *PIWIL1* is considered to be a highly expressed CT gene. Hypomethylation of the promoter DNA of *PIWIL1* can cause overexpression of CT genes [36]. However, to date, there are few reports on the function and mechanism of KK-LC-1 in human malignant tumours.

It is worth noting that our research findings can be regarded as a theoretical basis for immunotherapy and targeted therapy of different tumours involving KK-LC-1. Based on the Human Protein Atlas database (http://www.proteinatlas.org), *CT83* transcripts are expressed in various tumour cell lines, including gastric cancer, colorectal cancer, breast cancer, urothelial cancer, lung cancer, and cervical cancer. We speculate that *CT83* may be related to the body’s antitumour response. In normal tissues, the expression level of *CT83* is very low. However, when a tumour develops, the *CT83* expression level may increase as part of the immune response to the tumour. A higher *CT83* expression level indicates a stronger ability of the body to resist tumours and therefore a better prognosis. In studies of the early diagnosis of GC, researchers such as Futawatari found that a higher *CT83* expression rate can often be detected early [24]. Therefore, *CT83* can be used as a potential marker for the early diagnosis and treatment of GC.

Our study has some potential limitations. This is a single study with relatively few sample cases and few statistical analysis tools. Finally, some patients received postoperative chemotherapy or radiotherapy. Although the survival period was limited, the results did not consider the impact of these adjuvant therapies on prognosis. Therefore, further research and multi-angle studies are needed to explore KK-LC-1 expression in GC, and the clinical efficiency of KK-LC-1 needs to be evaluated in a wider range of patients. In summary, our project indicates that KK-LC-1 protein expression in GC is higher than that in neighbouring tissues. High levels of KK-LC-1 protein expression are associated with longer overall survival in GC. KK-LC-1 is a good biomarker for patients with GC.

## Conclusions

In summary, our project indicates that KK-LC-1’s protein expression in GC is higher than that in neighboring tissues. High levels of KK-LC-1 protein expression are associated with longer overall survival in GC. KK-LC-1 is a good biomarker for patients with GC.

## Data Availability

The datasets used and/or analysed during the current study are available from the corresponding author on reasonable request.
